# New perspectives on the regulation of type II inflammation in asthma

**DOI:** 10.12688/f1000research.11198.1

**Published:** 2017-06-28

**Authors:** Mireya Becerra-Díaz, Marsha Wills-Karp, Nicola M. Heller

**Affiliations:** 1 Department of Anesthesiology and Critical Care Medicine, Johns Hopkins University School of Medicine, Baltimore, MD, 21205, USA; 2 Department of Environmental Health and Engineering, Johns Hopkins Bloomberg School of Public Health, Baltimore, MD, 21205, USA

**Keywords:** asthma, Th2 inflammation, cytokines, alarmins, cellular metabolism, signalling, allergic inflammation

## Abstract

Asthma is a chronic inflammatory disease of the lungs which has been thought to arise as a result of inappropriately directed T helper type-2 (Th2) immune responses of the lungs to otherwise innocuous inhaled antigens. Current asthma therapeutics are directed towards the amelioration of downstream consequences of type-2 immune responses (i.e. β-agonists) or broad-spectrum immunosuppression (i.e. corticosteroids). However, few approaches to date have been focused on the primary prevention of immune deviation. Advances in molecular phenotyping reveal heterogeneity within the asthmatic population with multiple endotypes whose varying expression depends on the interplay between numerous environmental factors and the inheritance of a broad range of susceptibility genes. The most common endotype is one described as “type-2-high” (i.e. high levels of interleukin [IL]-13, eosinophilia, and periostin). The identification of multiple endotypes has provided a potential explanation for the observations that therapies directed at typical Th2 cytokines (IL-4, IL-5, and IL-13) and their receptors have often fallen short when they were tested in a diverse group of asthmatic patients without first stratifying based on disease endotype or severity. However, despite the incorporation of endotype-dependent stratification schemes into clinical trial designs, variation in drug responses are still apparent, suggesting that additional genetic/environmental factors may be contributing to the diversity in drug efficacy. Herein, we will review recent advances in our understanding of the complex pathways involved in the initiation and regulation of type-2-mediated immune responses and their modulation by host factors (genetics, metabolic status, and the microbiome). Particular consideration will be given to how this knowledge could pave the way for further refinement of disease endotypes and/or the development of novel therapeutic strategies for the treatment of asthma **.**

## Introduction

Asthma is a phenotypically heterogeneous inflammatory disease of the lungs generally characterized by airflow obstruction and airway inflammation. Advances in molecular phenotyping reveal heterogeneity within the asthmatic population with multiple endotypes ^1– 3^ whose varying expression depends on the interplay between numerous environmental factors and the inheritance of a broad range of susceptibility genes. Patients with a type-2-high profile are found in half of the mild asthmatic patients and are reported to be responsive to steroids ^3, 4^. The type-2-high asthma endotype is one of the most consistent endotypes to emerge ^3– 9^. This common subtype of asthma is characterized by the release of signature cytokines interleukin (IL)-4, IL-5, and IL-13 from cells of both the innate and the adaptive immune systems. These type-2 cytokines are targets for pharmaceutical intervention, and a number of therapeutic options are under clinical investigation for asthma. These will be discussed in detail below. The identification of multiple endotypes has provided an explanation for the observations that therapies directed at these typical T helper type-2 (Th2) cytokines and their receptors have often fallen short when they were tested in a diverse group of asthmatic patients without first stratifying based on disease endotype or severity. The stratification of patients in clinical trials based on specific endotypes has lent support for continued efforts to modify IL-4 and IL-13 pathways in type-2-high asthma, yet additional variation in treatment response is still apparent. These results suggest that regulation of type-2 cytokine production and responsiveness is more complex than was previously appreciated and that other genetic/environmental factors may be contributing to the diversity in drug response. As many ongoing efforts are designed to target the type-2 immune response, it is critical to fully understand the intricacies of regulation of this arm of the immune response. In this review, we will discuss new insights into the mechanisms by which IL-4- and IL-13-mediated inflammation is initiated, enhanced, perpetuated, or inhibited, with a focus on new players that modulate IL-4 and IL-13 responses. Although we will touch briefly on IL-5 as an additional important Th2 cytokine in promoting the allergic inflammatory response, we will focus our comments here on IL-4 and IL-13. We will highlight how some of the new pathways that influence IL-4 and IL-13 responses might potentially be harnessed for therapeutic benefit to downregulate type-2 immune responses in type-2-high asthmatics.

## Role of canonical Th2 pathways in allergic inflammation

The Th2 immune response is characterized by the expansion of CD4 ^+^ T cells producing the prototypical Th2 cytokines IL-4, IL-13, and IL-5. Although IL-9 was originally thought to be produced by Th2 cells, more recent studies suggest that it is primarily produced by a unique CD4 ^+^ T cell subset referred to as Th9 ^10, 11^. More recent studies show that other cell types such as type-2 innate lymphoid cells (ILC2s), mast cells, and eosinophils also produce these type-2-associated cytokines. These cytokines act on multiple cell types and trigger the hallmark features of a type-2 immune response, including the synthesis of immunoglobulin (Ig)E, eosinophilia, mast cell activation, mucus cell hyperplasia, and macrophage polarization. Although a type-2 immune response is critical in host defense against helminth infections, inappropriate activation of type-2 responses against otherwise innocuous antigens leads to allergic responses in different barrier organs, such as the skin, gut, and lungs.

Aberrant production of the prototypical type-2 cytokines IL-4 and IL-13 has long been associated with the pathogenesis of allergic disorders ^12, 13^. The overproduction of mucus resulting in airway obstruction in asthma is an effect of IL-4/IL-13 action on goblet cells, inducing mucin gene expression, hyperplasia, and hypertrophy ^14^. IL-4 and IL-13 also have direct effects on airway smooth muscle cells that may explain the hypercontractility of these cells in the airways of asthmatics ^15^. IL-4 and IL-13 also induce chemokine release from airway smooth muscle cells ^16^. The activity of IL-4 and IL-13 on the structural cells of the lung such as vascular endothelial cells and airway epithelial cells elicits the expression of adhesion molecules, chemokines, and transforming growth factor (TGF)-β production. These molecules direct the influx of circulating inflammatory cells, such as eosinophils, basophils, and others, to the lung tissue and airway lumen and induce lung tissue remodeling. Neutrophils also migrate into the lungs during allergic inflammation, but they are particularly elevated in patients with severe asthma. These patients are different from the type-2-high endotype and their disease is believed to be driven by Th17-mediated inflammation. As such, they are also typically unresponsive to the IL-4-/IL-13-directed therapies discussed in the previous section and to treatment with corticosteroids. IL-4 enhances the capacity of dendritic cells (DCs) to stimulate T cell secretion of Th2 cytokines, whereas IL-13 enhances the capacity of DCs to suppress T cell secretion of interferon (IFN)-γ in mice ^17^. Both IL-4 and IL-13 increase DC antigen uptake ^18, 19^ and cell migration into the lymph nodes where they prime naive T cells to differentiate into Th2 cells ^20^. B cell class switching to production of IgE is driven by IL-4 ^21, 22^ and by IL-13 in human but not in mouse B cells, which lack IL-13 receptor α1 (IL-13Rα1) ^23^. Fibroblast responses to IL-4 and IL-13 contribute indirectly to the lung remodeling that occurs in chronic asthma by eliciting the production of adhesion molecules and chemokines as well as cytokines that then stimulate epithelial cells to secrete TGF-β1 ^24^.

The eosinophilia observed in asthma and allergic diseases is partially controlled by the Th2 cytokine IL-5. IL-5 is critical for the genesis (in the bone marrow) ^23^, growth ^24, 25^, and survival of eosinophils. Eosinophils are a key target of therapeutic strategies in the treatment of allergic disease because of their ability to cause tissue damage and inflammation following the activation and release of a toxic mixture of their different granule proteins in inflamed tissue. IL-5 acts on eosinophils by binding to its receptor (IL-5R), which is a heterodimer composed of two chains: one α subunit (which binds IL-5) and one β subunit (which is implicated in signal transduction) ^25^. Accordingly, blocking IL-5 with antibodies has been useful at lowering peripheral and sputum eosinophil counts in asthmatics. While several studies indicate that anti-IL-5 treatment had no effect on improvement in quality of life measures in asthmatics compared to placebo controls ^26^, others have demonstrated that patients have fewer exacerbations concomitant with reduction in blood eosinophil counts ^27^. Beyond blocking IL-5, blocking the IL-5 receptor has also been an important therapeutic target against asthma. Currently, two anti-IL-5 antibodies are FDA approved for use in asthmatics older than 12 years of age ^27^, and a third antibody, which binds the IL-5Rα, is now in phase III clinical trials ^28^. However, as mentioned above, blocking IL-5 alone is not sufficient to decrease asthma severity. Future trials combining the blockade of IL-5/IL-5R together with other therapies will provide more robust strategies to improve quality of life in asthmatic patients.

Lung macrophages (alveolar and interstitial macrophages) are critical regulators of lung homeostasis and the inflammatory response to inhaled allergens ^29^. Similar to T cells, lung macrophages that encounter IL-4 and IL-13 polarize into a distinct phenotype, formerly known as “alternatively activated” or M2 macrophages. M2 macrophages express arginase I (ARG1) and other hallmark M2 gene products, such as YM1 and Found in Inflammatory Zone (FIZZ)1 in mice ^30^. This is in contrast to Th1 cytokines, viruses, and microbes that induce skewing to M1 macrophages and that express inducible nitric oxide synthase (iNOS), IL-1β, tumor necrosis factor (TNF)-α, and other pro-inflammatory mediators ^31^. Although both M1 and M2 macrophages are found in the lung during the course of allergic inflammation, M2 macrophages are increased in the lungs of asthmatics compared to healthy controls, and their abundance correlates with declines in lung function ^32– 38^. Mouse models of allergic inflammation recapitulate the observations in humans ^39– 42^. Adoptive transfer experiments revealed that the transfer of IL-4Rα-sufficient macrophages, but not IL-4Rα-deficient macrophages, into RAG2 ^–/–^ mice was sufficient for the development of allergen-driven lung eosinophilia ^40^.

M2 macrophages are thought to promote allergic lung inflammation through the secretion of angiogenic and pro-fibrotic factors, including vascular endothelial growth factor, insulin-like growth factor (IGF)-I, TGF-β1, matrix metalloproteinases (MMPs), acidic mammalian chitinase (AMCase), BRP-39/YKL-40 ^43^, and FIZZ1 ^44– 46^, that have the potential to promote lung remodeling. M2 macrophages also produce many inflammatory cell-recruiting chemokines (CCL11, 24, and 26, YM1, and others) that recruit eosinophils, basophils, T cells, and other immune cell types to the inflamed lung. Lastly, M2 macrophages have immunomodulatory properties also through direct and indirect effects on T cells, airway epithelium, and DCs ^47– 49^. Since alveolar macrophages are one of the “first responders” to inhaled allergens and owing to their key role in immunomodulation and maintaining homeostasis, modifying their phenotype and function is an attractive concept for intervention.

In the section below, we will describe how IL-4 and IL-13 regulate most of the features of allergic inflammation through their interaction with the IL-4/IL-13 receptor complexes and downstream signaling in multiple cell types. We will also highlight how new insights into these processes could provide opportunities for the development of therapeutic intervention in asthmatics.

## IL-4 and IL-13 cytokine receptor regulation

IL-4 and IL-13 are functionally and structurally related cytokines that have both overlapping and unique biological responses owing, in part, to the utilization of a combination of shared and unique receptors ^12, 50, 51^. IL-4 is recognized by two types of membrane receptors, type I (IL-4Rα and the common gamma-chain [γc]) and type II (IL-4Rα and IL-13Rα1), while IL-13 is recognized only by the type II IL-4 receptor. IL-13 is also bound with incredibly high affinity by IL-13Rα2, which exists in membrane-bound and soluble forms. IL-4 promotes and maintains the polarization of Th2 cells through ligating the type I IL-4R ^52^, whereas IL-13 has no effect on T cells because of their lack of IL-13Rα1 expression. On the other hand, IL-13 is thought to be more important in the induction of the physiological aspects of allergic asthma, including airway hyper-responsiveness (AHR), mucus hypersecretion, airway smooth muscle alterations, and subepithelial fibrosis, than IL-4 ^52, 53^. The exact molecular mechanism(s) by which these two cytokines regulate distinct features of the allergic response and how their contributions, either individually or in concert, induce the pathophysiological manifestations of disease remain to be fully uncovered. Recent discoveries of differences in utilization/assembly and affinity of IL-4 and IL-13 for the type-2 receptor and the regulation of the receptors along with the discovery of unique ILC2s which preferentially produce IL-13, and not IL-4, are beginning to shed light on these mysteries ^12^. Moreover, the identification of new cellular and molecular pathways affecting the regulation of IL-4/IL-13 receptor chains is painting a very complex picture in which a series of intricate feedback loops serve to keep the expression of type-2 inflammation in check. Understanding this delicate balance and how it is disturbed in asthma may inform the development of novel therapeutic approaches for the treatment of asthma.

### IL-4Rα

Given the importance of IL-4Rα in triggering Th2 inflammation ^54, 55^ and the strong association between polymorphisms in this gene and asthma risk ^56– 58^, understanding the mechanisms by which IL-4Rα expression is controlled is critical. In this regard, recent studies suggest a role for STUB1 (STIPI homology and U-box-containing protein 1), a chaperone-dependent E3 ubiquitin ligase ^59^ which interacts with IL-4Rα and targets it for proteasomal degradation to regulate signaling. STUB1-deficient mice show spontaneous airway inflammation, alternative M2 activation, and increased serum IgE levels, which was correlated with increased IL-4Rα expression. Interestingly, STUB1 mRNA levels are up-regulated in the airways of subjects with asthmatic and chronic obstructive pulmonary disease (COPD), suggesting that elevation of STUB1 expression may serve as a possible feedback mechanism in an effort to dampen IL-4Rα signaling ^60^. The development of strategies to modulate STUB1 activity may represent a promising approach for targeting IL-4-/IL-13-dependent allergic inflammation.

Several therapeutic strategies aimed at blocking IL-4Rα-initiated signaling have been attempted. These approaches include treatment with a variant IL-4R protein and with several individual monoclonal antibodies directed against IL-4Rα. First, a variant of the IL-4 protein, pitrakinra, that contains two amino acid changes that allow it to bind the IL-4Rα-chain, without allowing it to complex with either the γC or the IL-13Rα1 chains, has shown some promise. In an allergen challenge study, treatment with nebulized pitrakinra resulted in a decrease in the late-phase allergic response measured by FEV1 ^61^. A larger follow-up study of symptomatic moderate-to-severe adult asthmatics using corticosteroids revealed that although there was no therapeutic benefit for the entire population treated with pitrakinra compared to placebo, there was a significant reduction in asthma exacerbations in a pre-specified subset of subjects with high blood eosinophil counts ^62^. A later study revealed a pharmacologic interaction between therapy and variation (IL4RA Q576R) within the gene encoding the IL-4Rα chain ( *IL-4RA*), identifying an asthma subgroup that was more responsive to pitrakinra ^63^. Another strategy to block IL-4Rα signaling has been to treat with humanized high-affinity monoclonal antibodies directed against IL-4Rα (AMG-317, dupilumab). A randomized controlled phase IIA trial showed that treatment with dupilumab (SAR231893/REGN668, NCT01312961) prevented asthma exacerbations when long-acting β-agonists and inhaled corticosteroid were withheld in patients with moderate-to-severe asthma as compared to placebo ^5^. Phase III studies are underway which should provide more definitive information regarding the efficacy of blocking the IL-4Rα in asthma.

### IL-13Rα1

As discussed above, the role of IL-13Rα1 in mediating Th2 inflammation such as in AHR, mucus production, and fibrosis is well known ^64, 65^. IL-13Rα1 ^–/–^ mice did not develop AHR nor mucus production and had decreased IgE titers in a schistosomal egg antigen-induced model of allergic lung inflammation ^66^. Similarly, challenging the IL-13Rα1 ^–/–^ mice with IL-13, IL-4, or OVA resulted in no mucus production and protection from AHR ^64^. These studies demonstrate the critical role of IL-13Rα1 in the generation of AHR and mucus production. The development of the fibrotic response is also dependent on IL-13Rα1 in both the schistosomal and the allergic lung inflammation models of Th2 inflammation ^64, 66^. In response to worm or allergen challenge, IL-13Rα1-deficient animals had decreased expression of characteristic matrix remodeling genes and collagen deposition in their livers ^66^ and profibrogenic mediators such as TGF-β in their lungs ^64^. However, recent studies highlight a protective role for IL-13Rα1 in regulating fibrosis in a bleomycin-induced injury murine model. Karo-Atar *et al*. demonstrated that IL-3Rα1 deficiency resulted in a dysregulation in homeostasis and increased fibrosis due to exaggerated tissue repair ^67^. Although bleomycin-elicited lung inflammation and injury is not a typical allergic inflammatory response, these results suggest a role for the IL-13Rα1 as a sink for excess cytokine during the wound-healing response. This role could also be important in allergic inflammation, since IL-13Rα1-deficient mice have increased circulating IL-13 and soluble IL-13Rα2 ^64^. Another interesting study demonstrated that the integrin Mac-1 binds to IL-13Rα1 to downregulate macrophage IL-13 signaling, M2 polarization, and foam cell formation ^68^. Macrophages from the Mac-1-deficient mice had elevated M2 polarization, even in the absence of IL-13. Whether low Mac-1 expression could contribute to the abundance of M2 macrophages in asthmatic lungs is unknown. This work suggests that the interaction between Mac-1 and IL-13Rα1 may be exploited for the regulation of IL-13 signaling and M2 polarization of macrophages in the lung.

Because IL-13R (type II IL-4R) mediates the effects of IL-13 on structural (non-hematopoietic) cells, the clinical efficacy of a number of antibodies directed against IL-13 (IMA-638, IMA-026) has been evaluated in asthma. The results have been mixed. First, Wyeth evaluated the efficacy of two separate humanized anti-IL-13 IgG1 monoclonal antibodies (IMA-638 [NCT00339872]; IMA-026 [NCT00725582]) in asthma clinical trials. Interestingly, after allergen exposure, only treatment with IMA-638, which recognizes the IL-13 epitope that interacts with IL-4Rα, significantly reduced both the early and the late-phase asthmatic response. On the other hand, IMA-026, which binds to the epitope on IL-13 that is critical for binding to both IL-13Rα1 and IL-13Rα2, efficacious ^69^. Similarly, a more recent clinical trial utilizing another anti-IL-13 IgG1 monoclonal antibody (GSK679586, GlaxoSmithKline), which interferes with binding of IL-13 to both IL-13Rα1 and IL-13Rα2, did not result in any benefit in severe asthmatic patients over placebo controls ^70^. Thus, it appears that blocking the interaction of IL-13 with IL-4Rα may be more effective than inhibiting binding to either IL-13Rα1 or IL-13Rα2.

Based on the inconsistent results of the previous studies, a study was designed to compare the effects of blocking IL-13 in asthma patients who were pre-identified as exhibiting an IL-13 signature *in vivo* ^70^. In this randomized, double-blind trial of asthmatic patients with poorly controlled disease, patients were stratified into either Th2-high or -low subgroups based on their baseline Th2 status (IgE levels, blood eosinophil counts, and periostin) and treated subcutaneously with either placebo or 200 mg of the humanized IgG4 anti-IL-13 monoclonal lebrikizumab (Genentech, NCT00930163). Even in the presence of steroid treatment, lebrikizumab treatment resulted in an improvement in FEV1 in patients with a Th2-high phenotype but had no significant impact on FEV1 in patients with a Th2-low phenotype. These results demonstrate that blocking IL-13 in patients with a documented IL-13 signature may provide significant clinical benefit. As steroid treatment may have complicated the interpretation of the effects of IL-13 inhibition in the original study, a subsequent phase II randomized, double-blind, placebo-controlled study of lebrikizumab (NCT00971035) in asthmatic patients not receiving inhaled corticosteroids was conducted ^71^. In this study, lebrikizumab treatment did not show significant improvement in disease (pre-bronchodilator FEV1 values), despite effectively inhibiting Th2 biomarkers (IgE levels and exhaled nitric oxide [FeNO]). Surprisingly, these results suggest that IL-13 blockade may be effective in the context of steroid treatment but may not block disease in patients with mild disease (non-steroid dependent). Phase III studies are underway which should provide further clarification of the potential utility of IL-13 blockade in patients with uncontrolled asthma.

### IL-13Rα2

IL-13Rα2 was previously described as a decoy receptor that counteracted IL-13-mediated signaling ^72, 73^ owing to its negligible cytoplasmic domain. Although IL-13Rα2 does not have a signaling domain, recent studies have shown that IL-13Rα2 engages with TMEM219 and that this interaction also contributes to the decoy function of IL-13Rα2 ^74^. However, several lines of evidence suggest that the role of IL-13Rα2 in regulating inflammation may be more complex. For example, Fichtner-Feigl *et al*. reported that signaling through this receptor was involved in TGF-β1 production ^75^ and activation of the mitogen-activated protein kinase (MAPK) pathway ^76^. Consistent with a role for IL-13Rα2 in promoting allergic inflammation, Chen *et al*. reported that allergen-driven AHR and eosinophilic inflammation were attenuated, rather than enhanced, in IL-13Rα2-deficient mice ^77^. Normal allergic inflammatory responses were restored by lung epithelial overexpression of membrane IL-13Rα2 in IL-13Rα2-deficient mice, suggesting that membrane-bound and soluble forms of IL-13Rα2 may mediate different functions. Supporting a positive role for IL-13Rα2 in asthma is the recent finding that sputum levels of IL-13Rα2 are associated with poor lung function, Th2 cell gene expression, and airway obstruction in the airways of asthmatics ^78^. Collectively, these studies suggest that IL-13Rα2 is an important regulator of IL-13-mediated responses: the directionality of its effects are likely dependent upon whether it is found in a soluble or membrane-bound form. Lastly, as will be discussed below, IL-13Rα2 has been shown to have other binding partners besides IL-13, such as chitinase 3-like 1 (Chi3l1) ^74^. The complex role of IL-13Rα2 in regulating IL-13-mediated signals suggests that approaches to inhibit IL-13 could be a double-edged sword depending on whether or not they impact the ability of IL-13Rα2 to bind IL-13, which might result in the loss of the inhibitory functions of IL-13Rα2.

## New twists on IL-4 and IL-13 signaling pathways

Canonical signaling of IL-4 and IL-13 results in signal transducer and activator of transcription (STAT)6 phosphorylation and transcriptional activation of STAT6-dependent genes ^79^. However, STAT6signaling in response to IL-4 and IL-13 does not always explain all features of Th2-mediated inflammation. A second pathway that is differentially activated by IL-4 and IL-13 could explain some of the subtleties between the two cytokines. Specific activation of both IL-4R complexes by either IL-4 or IL-13 can also trigger insulin receptor substrate (IRS)-2 activation. IRS activation results in the growth-promoting, proliferative effects of IL-4 as well as activating the AKT target of rapamycin (TOR) pathway and gene expression downstream. However, the difference lies in the magnitude of IRS-2 activation by IL-4 compared to IL-13. IL-4 engagement of IL-4R type I triggers IRS-2 activation particularly robustly, significantly enhancing IL-4-induced responses such as M2 macrophage polarization over that elicited by IL-13 ^79, 80^. This was due to the presence of the γc chain in type I IL-4R complexes that is not found in the type II IL-4R. Thus, IRS-2 was concluded to be an amplifier of IL-4-induced M2 macrophage polarization, yet new evidence suggests a more complex role for the IRS-2 molecule. The absence of IRS-2 increased IL-4-induced M2 gene expression in murine bone-marrow-derived macrophages and stronger allergic lung inflammation *in vivo*. This novel regulatory role for IRS-2 was STAT6 independent ^81^ and suggests that IRS-2 activation may participate in a negative feedback inhibitory loop through the TOR complex to decrease IL-4 signaling. This will be discussed further in the metabolism section of this review.

In contrast, when IL-4-induced IRS-2 activation was prolonged through knockdown of the negative regulator suppressor of cytokine signaling (SOCS)1 *in vitro*, enhanced ** M2 polarization of human monocytes was observed ^82^. Furthermore, dysregulation of IRS-2 signaling in monocytes from allergic asthmatics was associated with increased M2 macrophage polarization owing to the lack of SOCS1 induction and increased SOCS3 expression ^82^. SOCS3 was also highly induced in M2 macrophages at the site of contact hypersensitivity (CHS) to control MMP-12 expression and CHS pathology ^83^. Taken together, differential expression of SOCS1 and SOCS3 in healthy versus inflammatory conditions suggests that an important balance exists between these molecules to maintain homeostasis in macrophages. A similar paradigm exists in T cells. SOCS3 and SOCS5 are mainly expressed in Th2 and Th1 cells, respectively, and reciprocally inhibit Th1 and Th2 differentiation ^84^. When dysregulation of the balance between different SOCS proteins occurs, pathological Th2 or M2 polarization results. Numerous examples of dysregulation of SOCS expression in eosinophils ^85^, airway smooth muscle ^86^, skin ^87^, and T cells ^88^ have been documented in allergic inflammation. Single nucleotide polymorphisms (SNPs) in the SOCS1 promoter are associated with adult asthma and total serum IgE in children ^89^. Hence, restoring SOCS function to ameliorate inflammatory conditions could be considered as a future therapeutic strategy for asthma, as it has been in the setting of autoimmunity ^90^.

## Downstream regulation of allergic inflammation

IL-4 and IL-13 activation of their receptors on a variety of structural and hematopoietic cells leads to the transcription of a wide variety of mediators, which mediate the pathophysiological manifestations of allergic disease, several of which have recently received considerable attention.

### Chitinases

Several chitinase (chitin-degrading enzymes) genes are induced by type-2 cytokines in humans and mice including AMCase, YKL-40 (Chi3l1), breast regression protein (BRP)-39, and YM1/2 in mice ^91^. ******** Chitin is a highly abundant polysaccharide in nature and is an essential component of many organisms that drive type-2 immune responses (fungi, arthropods [house dust mites, crabs, and shrimp], and parasites). The functions of chitin are highly dependent upon its size: chitin has been shown to induce eosinophilic lung infiltration ^92^ and elicit IL-33 production from epithelial cells ^93, 94^. The role of AMCase, the only IL-13-induced chitinase with chitinase enzymatic activity, in asthma has been conflicting ^95^. In contrast, many, but not all, studies suggest that YKL-40 is increased in severe asthma and/or neutrophilic asthma ^96^. Epithelial cells are thought to be the primary producers of YKL-40, and mechanical stress has been shown to be a trigger of YKL-40 secretion through an epidermal growth factor receptor (EGFR)- and MEK1/2-dependent pathway ^97^. YKL-40 has been reported to regulate a number of functions in the lung including IL-8 production ^98^, MUC5AC production ^99^, and the proliferation of bronchial smooth muscle cells ^100^. Recent studies demonstrated that the mouse homolog of YKL-40, Chi3l1, binds to and signals via IL-13Rα2. The same group later demonstrated that the membrane protein TMEM219 is a binding partner of IL-13Rα2. Interestingly, blockade of TMEM219 or IL-13Rα2 phenocopied one another in their ability to decrease Chi3l1-stimulated epithelial cell HB-EGF production, macrophage MAPK/ERK and protein kinase B (PKB)/AKT activation, oxidant-induced apoptosis, and lung injury. These studies demonstrate that an important regulatory loop exists among IL-13, Chi3l1, and IL-13Rα2, although the precise role of these interactions in asthma pathogenesis remains to be determined.

### FIZZ1

Although FIZZ1 ( *Retnla*) was identified as a marker of Th2 inflammation ^101, 102^, recent reports suggest the opposite. The increase in abundance of FIZZ1 in allergic diseases was interpreted as promoting Th2 responses ^101, 103^, yet Lee *et al*. demonstrated that overexpression of FIZZ1 did not induce alterations in lung histology and lung function. In contrast, *Retnla*-overexpressing mice exhibited a reduction in lung inflammation following allergen sensitization and challenge ^104^. This new evidence suggests a negative regulatory role for FIZZ1, agreeing with studies showing that mice lacking FIZZ1 developed exacerbated lung inflammation after *Schistosoma mansoni* (Sm) eggs challenge compared with their wild-type counterparts ^105^. Therefore, the elevated expression of FIZZ1 appears to mediate negative regulation of Th2 inflammation in the lung.

## New pathways regulating type-2 cytokine production

Activation of the Th2 pathway is initiated from a complex interaction between the innate and adaptive immune responses. Although the critical role of antigen-presenting cell interactions with CD4 ^+^ T cells in the differentiation of naïve T cells into type-2 cytokine-producing cells has long been appreciated,new information is emerging regarding the complex regulation of DC–T cell interactions that result in Th2 immune responses. Moreover, our understanding of the role of epithelial-derived cytokines in the direction of Th2 differentiation and in the regulation of non-T cell sources (ILC2s) of type-2 cytokines is rapidly expanding.

### DC–T cell interactions

Antigen presentation to naïve T cells is an essential step in the development of adaptive immune responses. Recent studies have identified a member of the tumor-associated macrophage (TAM) family receptor tyrosine kinase, TYRO3, as an important negative regulator of DC–T cell interactions and consequently the magnitude of type-2 immune responses ^106^. Specifically, TYRO3-deficient mice or neutralization of its orthologue in human DCs resulted in the enhancement of type-2 immunity. Interestingly, the TYRO3 agonist protein S1 (PROS1) is specifically induced in CD4 ^+^ Th2 cells by IL-4. PROS1, in turn, ligates the rheostat TYRO3 on PDL2 ^+^ DCs to limit the intensity of type-2 responses. Importantly, multiple intronic variants in TYRO3 were associated with asthma ^106^. Modulation of this self-limiting process intrinsic to type-2 immunity provides a novel opportunity to inhibit allergic responses.

Little is known about the signals from DCs that drive Th2 immune responses. New studies show that specific subsets of DCs may contribute to this process. Gao *et al*. have shown that PDL2 ^+^ DCs are able to enhance proliferation and cytokine production by effector and memory CD4 ^+^ T cells, but not in naïve cells, compared with PDL2 ^–^ DCs ^107^. Other studies using *in vivo* depletion approaches have demonstrated an important role for CD301b ^+^ but not for CD207 ^+^ dermal DCs in driving Th2 differentiation in both OVA-specific transgenic CD4 ^+^ T cells and during infection with *Nippostrongylus brasiliensis* ^108^. These novel studies suggest that depletion/inhibition of specific DC subsets is a potential therapeutic approach to suppress the initiation of Th2 immune responses.

### T cell receptor regulation

T cells play a determinant role in adaptive responses. They are able to recognize an infinite diversity of antigens through the T cell receptor (TCR) with concomitant activation of the T cell ^109^. As mentioned above, different polarized T helper cell environments participate in the different asthma phenotypes, highlighting the importance of T cell subset differentiation in allergic inflammation. Few T cell intrinsic factors have been identified which govern Th2 differentiation. Newly described mechanisms have been identified that downregulate TCR signaling specifically in Th2 cells. DENND1B, a guanine nucleotide exchange factor ^110^, downmodulates TCR expression in Th2 cells. Yang *et al*. demonstrated that *in vitro*-differentiated Dennd1b ^–/–^ Th2 cells have increased TCR-mediated responses when compared to Dennd1b ^+/+^ Th2 cells without changes in Th1 or Th17 responses. This specific effect on Th2 cells may be due to a delayed surface TCR downmodulation upon activation, resulting in increased TCR signaling. This appears to have direct relevance to asthma, as SNPs at the human DENND1B locus have been associated with increased Th2 responses and asthma in young children ^111^.

### E3 ubiquitin ligase regulation of Th2 cells

Recent studies suggest that E3 ubiquitin ligases are critical regulators of T cell activation and cytokine production. For example, overexpression of *Grail*, an E3 ubiquitin ligase, has been associated with suppressed IL-2 and IL-4 production in T cells ^112^. Mechanistically, *Grail* regulates Th2 cytokine production by interacting with STAT6, targeting it for ubiquitination and degradation. Accordingly, *Grail*-knockout mice are more susceptible to allergen-driven asthma ^113^. Another E3 ubiquitin ligase, casitas B cell lymphoma (Cbl)-b, which is involved in regulating CD28 signal strength during TCR ligation, has also been shown to be important in the regulation of allergic inflammation ^114^. Cbl-b-deficient mice have enhanced Th2 cytokine production and delayed resolution of allergen-induced airway inflammation. Interestingly, no changes in IgE levels were noted. Modulation of this class of molecules may provide a unique approach to asthma therapy.

### Th17 pathway regulation of Th2-mediated inflammation

In addition to the prototypical Th2 cytokines, other previously unrelated cytokines, such as IL-17, have now been demonstrated to modulate Th2 immune responses. Understanding the molecular mechanism(s) of IL-17’s modulatory role in Th2 responses is of interest, since “mixed” Th2/Th17 T cell populations are present in some asthmatic patients *in vivo* ^115, 116^. Co-exposure of mice to IL-13 and IL-17 enhanced all aspects of the allergic phenotype compared to IL-13 treatment alone ^115^. This was associated with an IL-17-mediated increase in IL-13-induced STAT6 activation in both mouse fibroblasts and primary human nasal epithelial cells (NECs) ^117^. Conversely, specific suppression of Th2 cytokines in the house dust mite (HDM) model of allergic asthma enhanced Th17 responses, and “Th2-high” and “Th17-high” disease was mutually exclusive in some asthmatic patients ^118^. These contradictory studies highlight that the exact mechanism(s) dictating the balance between Th2 and Th17 responses remain unclear. Careful immunophenotyping of patients prior to IL-17- or IL-4-/IL-13-based therapies will be essential, as disturbance of the Th2/Th17 balance may worsen the disease in some individuals. Blockade of IL-17RA with brodalumab, a human anti-IL-17RA monoclonal antibody, in subjects with inadequately controlled moderate-to-severe asthma taking regular inhaled corticosteroids did not have a significant impact on asthma. However, there was a positive effect on the asthma control questionnaire score in the high-reversibility subgroup (post-bronchodilator FEV1 improvement >20%) ^119^. As the significance of this effect is currently not known, further studies are required to unravel the complexities of the contribution of IL-17 to asthma pathogenesis.

### IL-31

IL-31 is constitutively expressed by non-hematopoietic cells such as the lung epithelium and is also selectively produced by activated CD4 ^+^ T cells skewed towards Th2 cytokine production ^120^. IL-31RA is a gp130-like type-1 cytokine receptor that pairs with OSMRB to form a functional signaling receptor for IL-31. IL-4 and IL-13 increase the expression of the membrane-bound form of IL-31RA through the type II IL-4R, altering IL-31-mediated signaling in macrophages. IL-31 has been shown to be elevated in Th2-mediated diseases such as atopic dermatitis ^121^ and is positively associated with asthma severity ^122^. Consistent with a role for IL-31 in asthma, Yu *et al.* showed that SNPs in IL-31 were significantly correlated with total serum levels of IgE in patients with asthma ^123^. Despite the preponderance of evidence suggesting that IL-31 promotes type-2 inflammation, Perrigoue *et al*. showed that IL-31R activation limited the magnitude of Th2 cytokine-dependent inflammatory responses to intestinal helminth infection ^124^. Taken together, these studies highlight important interactions between Th2 cytokines and IL-31 signaling pathways; however, further studies are needed to delineate the exact role that IL-31 plays in mediating type-2 immune responses and whether this will be a useful target for therapy in type-2-high asthmatics.

### Alarmins as initiators of type-2 inflammation

Although Th2 cytokine production has long been recognized as a pivotal contributor to allergic inflammation, the mechanisms initiating Th2 cell differentiation and cytokine production has long eluded investigators. Recent studies have identified several epithelial-derived molecules such as thymic stromal lymphopoietin (TSLP), IL-25, and IL-33 as early initiators of Th2 inflammation in both mice and humans. These cytokines have been described as "epithelial-derived alarmins" that activate and potentiate the innate and humoral arms of the immune system in response to cellular damage. Each of the three epithelial-derived alarmins has been implicated in the pathophysiology of allergic asthma. A better understanding of the roles that these epithelial-derived alarmins play in disease and how they influence airway immune responses may allow the development of novel therapeutics for asthma treatment.

***TSLP.*** The role of TSLP in allergic lung inflammation has garnered considerable interest. Early studies of mice overexpressing TSLP in airway epithelial cells resulted in spontaneous lung inflammation, enhanced eosinophilia, goblet cell metaplasia, perivascular fibrosis, and AHR ^125^. Moreover, Han and colleagues found that overexpression of TSLP in the lung induced an alternatively activated phenotype in pulmonary macrophages and increased BAL cell recruitment ^126^. Support for a role for TSLP was confirmed in studies utilizing *Tslpr* ^–/–^ mice in a model of lung inflammation ^127^. Interestingly, TSLP effects were not direct but were dependent on IL-13 ^126^. Consistent with the mouse studies, therapeutic antibody blocking of TSLP resulted in a reduction of lung eosinophils in a cynomolgus monkey model of allergic lung inflammation ^128^. TSLP also synergizes with IL-25 and IL-33 to promote inflammation and lung fibrosis induced by *Schistosoma*. Only antibody blockade of all three cytokines suppressed Th2-mediated fibrosis, although this approach did not work in a model of chronic HDM-induced allergic lung inflammation ^129^. These results suggest that fibrosis may be induced in the lung through multiple pathways. TSLP seems to play a more important role in the gut, where it is constitutively expressed in both mice and humans. In the gastrointestinal tract, TSLP is not only an important enhancer of inflammatory Th2 responses ^130– 132^ but also a key player in the maintenance of homeostasis by controlling inflammatory responses against parasites ^133– 137^ and Th1 and Th17 responses ^138, 139^. Therefore, the function of TSLP is complex, both promoting and controlling inflammation in a tissue-dependent manner. Support for a role for TSLP in human disease has recently been provided in a study of a human anti-TSLP monoclonal antibody (AMG 157), which binds TSLP and prevents receptor interaction. In a double-blind, placebo-controlled study, AMG 157 attenuated both early and late allergen-induced bronchoconstriction and indices of airway inflammation in allergic asthmatic subjects ^140^. Although these results are promising, further studies are required.

***IL-25.*** IL-25, also named IL-17E, is a cytokine able to enhance Th2 immune responses. It was first described as a product of “highly polarized” murine Th2 cells in the presence of IL-4/anti-IL-12 monoclonal antibodies ^141^. However, more recent studies indicate that IL-25 can be produced by a great variety of cells including eosinophils, basophils, mast cells, macrophages, epithelial cells, and Tuft cells, among others ^142^, highlighting its importance as an initiator of Th2 responses. For example, during *N. brasiliensis* infection, hyperplasia of Tuft cells—the only IL-25-releasing cell—occurs in the small intestine. A feed-forward system mediated through IL-25 release by Tuft cells activates ILCs to release IL-13, which in turn regulates the number of Tuft cells ^143^. Interestingly, Tuft cells express taste receptors such as bitter and umami receptors, which are G-protein-coupled receptors (GPCRs), that participate in the release of acetylcholine when activated ^144^. This has been associated with changes in nutrient and metabolite levels in the gut lumen, which could help Tuft cells detect danger signals, such as the presence of helminths ^145^, and initiate a protective Th2 immune response. The signaling and functional responses to helminths at the gut epithelial barrier show remarkable similarity to the lung in that bitter taste receptors and early alarmin signaling may work together to coordinate the immune response to inhaled antigens. Blockade of the IL-25 receptor, IL-17RA, in bronchial rings from donors with asthma significantly reduced acetylcholine-induced contraction compared to that seen in donors without asthma ^146^. One of the major triggers of asthma exacerbations, rhinovirus infection, was shown to induce significantly greater expression of IL-25 mRNA expression in bronchial epithelial cells from asthmatics compared to those in non-atopic, non-asthmatic healthy individuals ^147^. Although these studies suggested that blocking IL-17RA in asthma might be promising, studies with the anti-IL-17RA monoclonal antibody brodalumab have failed to show benefit in asthma ^119^, implying that blocking IL-25 alone is insufficient to modify disease.

***IL-33.*** Another alarmin that has risen to prominence in recent years is IL-33, a novel cytokine discovered almost 10 years ago ^148^. The importance of IL-33 in allergic disease was initially identified through a genome-wide association study (GWAS). The GABRIEL consortium, the largest GWAS meta-analysis involving more than 26,000 subjects, demonstrated significant association between asthma and a SNP (rs1342326) on chromosome 9 flanking the *IL33* gene. Polymorphisms in the *IL33* gene were among the top three SNPs to correlate with asthma ^149^. Similarly, strong associations between variants of the gene encoding the unique IL-33 receptor, suppression of tumorigenicity ( *ST2*) or *IL1RL1*, and asthma risk have been observed ^150^. The identification of IL-33 and its receptor as asthma susceptibility genes has opened up a fruitful new field of study that has exponentially increased our understanding of the mechanisms driving aberrant type-2 immune responses.

IL-33 has been shown to be an important regulator of type-2 cytokine production and the pathogenesis of allergic diseases. In several mouse models of asthma, IL-33 activates lung-resident ILC2s and initiates airway type-2 inflammation ^20, 151^. Similarly, administration of rIL-33 to mice through a variety of routes resulted in tissue eosinophilia, goblet cell hyperplasia, and elevations in type-2 cytokines ^152^. The timing of expression of IL-33 in the lung is thought to be critically important to the development of skewed Th2 immune responses. It has been reported that shortly after birth, ILC2s, eosinophils, basophils, and mast cells spontaneously accumulate in the developing lung in an IL-33-dependent fashion ^153^. Upon encounter with allergens during early lung development, the changes are further boosted, promoting Th2 cell skewing. Thus, enhanced neonatal Th2 cell skewing in response to exposure to inhaled allergens results from postnatal hyper-responsiveness of the IL-33 axis during a period of maximal lung remodeling.

IL-33 is a member of the IL-1 family of cytokines, which is constitutively expressed in the nucleus of a variety of cells in both mice and humans ^148^. Because of its localization within the nucleus, it is thought to function as a nuclear receptor, although its exact function in the nucleus is unknown. In addition to potential nuclear functions, IL-33 is released primarily from endothelial and epithelial cells and binds its functional receptor on a variety of cells, including mast cells, eosinophils, and ILC2s ^154^. Unlike other IL-1 family members, IL-33 does not require cleavage by caspase 1 for release from the cell or to initiate signaling via ST2 ^155^; however, it can be inactivated by either caspase-3 or caspase-7. On the other hand, cleavage by extracellular proteases (chymase and tryptase), commonly produced by inflammatory cells such as mast cells present during allergic reactions, results in three major forms of “mature” IL-33. These cleaved versions of IL-33 are up to 30-fold more potent as activators of ILC2s *ex vivo* compared with full-length human IL-33 ^156^.

Despite the importance of IL-33 to the initiation of an allergic response at mucosal surfaces, the molecular mechanisms responsible for its expression and extracellular release are poorly understood. IL-33 transcription is believed to be initiated from one of two non-coding exons that are associated with constitutive or induced production of IL-33 ^157^. A recent study suggested that the RNA-binding protein Mex-3B regulates IL-33 expression in airway epithelial cells via binding to the IL-33 3' UTR and increasing IL-33 expression by inhibiting miR-487b-3p-mediated repression of IL-33 ^158^. The importance of Mex-3B to the regulation of IL-33 in allergic inflammation is demonstrated in Mex-3B-deficient mice, which are protected against allergen-induced airway inflammation due to the induction of IL-33. Moreover, it was shown that inhalation of an anti-sense oligonucleotide targeting Mex-3B suppressed the development of allergic airway inflammation in mice.

As IL-33 lacks a traditional signal sequence ^159^, it was originally thought to be released primarily by damaged or necrotic cells ^160^. However, a more complex picture is emerging in which it can be released from both human and mouse living cells both constitutively and following exposure to a variety of stimuli ^161, 162^. Under homeostatic conditions, pools of IL-33 become active locally to sustain basal physiology by activating ST2 ^+^ cells. In response to a number of perturbations, such as allergen exposure, it can be induced through multiple pathways in the absence of cell death depending on the nature of the stimulus. For example, common allergens such as *Alternaria alternata* and HDM induce IL-33 secretion through a mechanism dependent on DUOX-1-mediated activation of the epithelial EGFR and the protease calpain-2 through a redox-dependent mechanism involving cysteine oxidation of the EGF and the tyrosine kinase Src ^163^. Consistent with the importance of this pathway to allergic disease, NECs from allergic asthmatic patients were shown to express more DUOX-1 and IL-33 compared to non-asthmatic individuals. Several studies also suggest that extracellular ATP can induce the secretion of IL-33 ^164^. Alternative splicing resulting in cytoplasmic IL-33 has also been proposed as a potential mechanism of extracellular release ^165^. Consistent with these findings, splice variants of human *IL33* have been recovered from primary cells; however, the physiological relevance of these variants is currently unknown ^166^. IL-33 is clearly a key player in the initiation of allergic inflammation, suggesting that it would be an excellent target for biologic therapy. However, as it also plays a role in other non-Th2-mediated inflammatory diseases ^167, 168^, a more detailed understanding of the plethora of IL-33’s actions is required prior to targeting it in allergic individuals.

## Impact of cellular metabolism on Th2-mediated inflammation

In recent years, the role of metabolism in regulating the function of immune and non-immune cells that are important in asthma has been uncovered. Balancing nutrient utilization and metabolism is critical for cells to meet energy needs required to survive, proliferate, and perform their specific functions (i.e. cytokine secretion). A variety of metabolic mediators such as arginine, extracellular lactate, and cyclic adenosine monophosphate (cAMP)/ATP have been implicated in various aspects of the asthmatic phenotype. Recent evidence suggests that the integration of these metabolic functions, sensing of the external nutrient milieu, and immune cell polarization occurs through the PI3K/AKT/mammalian TOR (mTOR) pathways ^169, 170^. While the importance of immune cell metabolic remodeling in different physiological settings is not fully understood, there is a growing realization that inappropriate metabolic function underlies many aberrant immune responses and that reprogramming cellular metabolism may provide a therapeutic avenue to alter immunity ^171, 172^.

### Arginine metabolism

Arginine is a key substrate in the citrulline–NO and tricarboxylic acid cycles. However, little is known about how mitochondrial arginine metabolism modulates inflammation. Elevated expression of the arginine-metabolizing enzymes iNOS and arginase were found in the airway epithelium of asthmatics. Xu *et al*. described a mechanistic role for arginine metabolism in asthma. Greater arginine flux increased oxidative metabolism in airway epithelium, resulting in lower STAT6 and hypoxia-inducible factor signaling, which are both important drivers of asthma ^173^. Arginase 1 is also a critical regulator of ILC2 metabolismin the lung. Elevated *ARG1* expression was noted specifically in ILC2s from the lung tissue of patients with COPD and idiopathic pulmonary fibrosis. Deletion of *Arg1* specifically in ILC2s resulted in the attenuation of type-2-mediated lung inflammation in mice due to diminished ILC2 proliferation and cytokine production. This work has shed new light on how arginine metabolism may influence allergic inflammatory responses ^174^. Since ILC2s are one of the major producers of the cytokines that initiate Th2 immune responses in allergic inflammation, the modulation of arginine metabolism may provide a promising avenue for the regulation of these low-frequency cells, thereby preventing the initiation of Th2 inflammation.

### Lactate

Recent studies have demonstrated that lactic acid produced by tumor cells induces an M2-like polarization with enhanced *Arg1* expression in murine TAMs compared with peritoneal macrophages ^175^. Similarly, a recent study by Selleri *et al*. demonstrated that the presence of lactate during the differentiation of human monocytes to DCs induced a M2 macrophage phenotype. These M2 macrophages induced Th2 polarization. This effect was due to a decrease in oxidative phosphorylation, lower basal respiration, and more polarized mitochondrial membrane potential as well as higher spare respiratory capacity ^176^. This lactate-induced M2 phenotype is consistent with the lactic acidosis observed in patients with severe asthma ^177, 178^ and the observation that asthmatics have more M2 macrophages in their lungs than do non-asthmatics ^38, 179^. Furthermore, lactic acid has been shown to delay NF-κB activation and suppress LPS-induced TNF-α secretion in monocytes, promoting an M2 macrophage phenotype ^180, 181^. However, opposite effects have been attributed to lactate, the salt form of lactic acid, in that it enhanced LPS-mediated inflammatory responses in macrophages ^182^. Recent studies have shown that lactic acid negatively regulates IL-33-mediated cytokine and chemokine production in murine bone-marrow-derived mast cells and primary human mast cells, resulting in the suppression of inflammation ^183^. Accordingly, lactic acid and lactate might be playing different roles in M2 polarization and asthma depending on the local pH. A better understanding of the conditions that induce either systemic or localized changes in airway pH may inform the development of more effective strategies to control asthma.

### mTORC

mTOR complex (mTORC)-activated pathways have emerged as critical regulatory nodes for the polarization of T cells ^184, 185^ and macrophages ^186^. The TORC1 pathway is crucial for the development of Th17 polarization in naïve murine T cells, but not the other Th profiles ^187^, linking the opposing regulation between Th2 and Th17 responses. This points to the mTOR pathway as a possible target for effective therapy to ameliorate Th2/Th17 mixed asthma phenotypes. Exploring the mTOR pathway for type-2-high asthmatics should be approached with caution, however, given the uncertainty surrounding equivocal data on the Th2/Th17 balance discussed above.

Regulation of macrophage polarization following the TORC1/2 dichotomy established for T cells has been an attractive concept. IL-4-dependent M2 macrophage differentiation is associated with mTORC2/STAT6 activation and increased fatty acid oxidation and oxidative phosphorylation ^188^. In contrast, constitutive activation of mTORC1 due to myeloid-specific deletion of its negative regulator tuberous sclerosis complex 1 leads to decreased M2 macrophage polarization by attenuating IL-4-induced AKT activation and M2 gene expression ^189, 190^. This correlates with the novel role of TORC1-activated p70S6K1 and GRB10 as key negative regulators of IL-4-induced IRS-2 signaling and M2 macrophage polarization ^191^. The balance between mTORC1 and mTORC2 in controlling macrophage polarization was also highlighted by studies in mice with a myeloid-specific deletion of rapamycin-insensitive companion of mTOR, a subunit of mTORC2. Lacking functional mTORC2 resulted in exaggerated responses to LPS and other TLR ligands and decreased M2 macrophage markers ^192^. Taken together, these studies highlight the importance of mTOR complexes in dictating M2 polarization. In addition, they point to possible targets, such as p70S6K or GRB10, that could be used therapeutically to downmodulate IL-4-mediated inflammatory responses and M2 macrophage polarization and to control type-2-high asthma.

### cAMP/ATP

cAMP also alters cellular phenotype. Bone-marrow-derived DCs from mice deficient in the production of cAMP triggered Th2 polarization of CD4 ^+^ T cells. These mice developed spontaneous elevations in total IgE, resulting in severe Th2-mediated lung inflammation. Exposure to the cell-permeable cAMP analog 8-CPT-cAMP (CPT) decreased their ability to induce Th2 responses ^193^. However, the role of cAMP in inflammation is more complex and could be cell specific. Mucus production by bronchial epithelial cells depends not only on IL-13 stimulation but also on signaling through the β2-adrenoceptor (β2AR). New research shows that epinephrine activation of β2AR and ERK1/2, JNK, and cAMP signaling is necessary for mucin production. These findings may explain the side effects of chronic β2AR agonist use in asthma ^194^.

In addition to cAMP, ATP has also been demonstrated to regulate inflammation. Through the utilization of a mouse model of OVA-induced lung inflammation, it was demonstrated that the P2X4 receptor (P2X4R) was upregulated in inflamed mouse lungs. Moreover, through binding to P2X4R, ATP enhanced the expression of α-smooth muscle actin ^195^ and proliferating cell nuclear antigen ^196^ in the lungs of allergically challenged mice. Overexpression of these two proteins is an indicator of cell proliferation in asthma. Furthermore, P2X4R antagonists decreased cell recruitment and mucus production in OVA-challenged mice. Taken together with ATP’s role in IL-33 release mentioned above, these studies suggest an important role for ATP in the regulation of airway inflammation and airway remodeling ^197^.

## The microbiome as a modifier of Th2 inflammation

There has been an increased appreciation of the role of the gut and lung microbiota in shaping the Th2 response in asthma. The beneficial effects of exposure to certain bacterial products at key times in immune system development have been demonstrated to reduce the development of asthma in epidemiological studies of infant breastfeeding, vaginal delivery, exposure to certain pets in the home ^198– 200^, the number of siblings in the home ^201^, and exposure to farm dust *in utero* ^202^. Early life antibiotic use ^203, 204^, lack of breastfeeding ^205^, or cesarean birth ^206^ are associated with an increased risk of developing asthma. While no specific pathogens have been associated with the protective effect of these exposures against disease, recent studies in human asthmatics have suggested that alterations in the diversity of the gut and lung microbiome may be associated with an increased risk of developing asthma ^207, 208^.

In a recent study in which the effects of the gut microbiome were investigated at the level of genera, the relative abundance of *Lachnospiraceae, Veillonella, Faecalibacterium,* and *Rothia* were found to be decreased in the gut in the first ** 100 days of life in children who later went on to develop atopic asthma ^209^. Of note, the differences in relative abundance of these genera were no longer present at 1 year of age. The contribution of these species to asthma pathogenesis was further demonstrated in an experimental mouse model. Asthma was induced in germ-free mice that were colonized with the gut microbiome of a child with severe atopic allergy and very low relative abundance of *Lachnospiraceae, Veillonella, Faecalibacterium,* and *Rothia.* The effect was reversed with supplementation with the four protective bacterial genera. If replicated, these findings would provide a strong basis for a causal link between these four bacterial genera and the development of atopic asthma. These results are consistent with multiple studies suggesting that there is a critical time period in early life when gut flora imbalances have the most influence on allergic inflammation ^209– 211^.

Studies in experimental animal models have shed some light on the potential mechanism(s) by which the shifts in phylogenic groups condition the immune system. For example, an intriguing study showed that the susceptibility of newborn mice to the development of HDM-driven BAL eosinophilia and AHR was associated with a lung microbial pattern dominated by *Firmicutes* and gammaproteobacterial phyla ^212^. Interestingly, over the first few weeks of life, the balance of lung phyla shifted towards a dominant *Bacteroidetes* pattern. This shift in the lung microbiota was associated with a reduction in responsiveness to allergen exposure and an increase in the population of PD-L1-dependent T-regulatory cells. Thus, these studies suggest that the establishment of a *Bacteroidetes*-dominated airway microbial pattern early in life is required to populate the lung with T-regulatory cells which provide sustained protection against the development of aeroallergen-induced airway inflammation.

Further support of the concept that gut colonization with *Bacteroidetes* is associated with protection against the development of the asthmatic phenotype was provided by a study in mice in which a high-fiber diet protected them from the aeroallergen-induced asthma phenotype, concomitant with a shift towards predominant gut colonization with *Bacteroidetes* ^212^. The protection afforded by the high-fiber diet was mediated via short-chain fatty acid-induced alterations in bone marrow hematopoiesis characterized by enhanced generation of macrophage and DC precursors and subsequent recruitment to the lungs of DCs with an impaired ability to promote Th2-cell differentiation. Mechanistically, the authors showed that the effects of one of the short-chain fatty acids (propionate) on the asthma phenotype were mediated via the GPCR 41 (GPR41). These three products of the fermentation of dietary fiber are absorbed by the host, where they circulate and communicate with the immune system by binding to host cell receptors. Indeed, short-chain fatty acids, such as butyrate and acetate, have been shown to regulate mucosal T-regulatory cell differentiation and function ^213^. In humans, decreased fecal short-chain fatty acids at 3 months of age have been observed in subjects who went on to develop atopy and wheeze at 3 years of age, and germ-free mice inoculated with the microbiome of a child with atopic wheeze had lower levels of the short-chain fatty acid butyrate than did control mice ^214^.

Taken together, these results suggest that diet and the gut microbiome shape the immunological environment in the lung and that a disrupted microbiota could possibly have detrimental effects. Importantly, these results suggest that microbiota-based interventions could be used to influence the likelihood of developing allergies. However, the timing of administration of such therapies is critical and would likely have to be given in the “window of opportunity” in early life to be effective. Further research is now required to fully dissect the role of the microbiome in disease and ultimately pave the way for the emergence of new therapeutic strategies in combating these conditions.

## Conclusion

**Figure 1.**
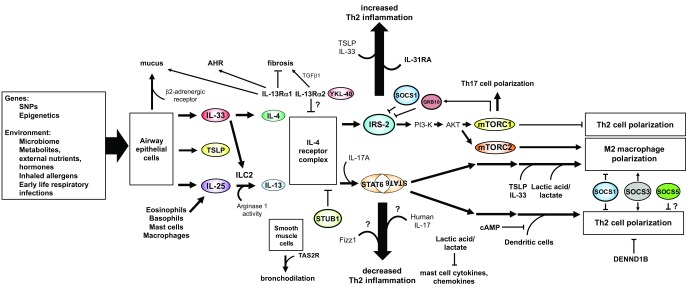
Summary of the canonical Th2 pathway and “new spins” on the pathways that lead to allergic inflammation. Newly appreciated modifiers of Th2 inflammation, such as alarmins, environmental factors, and G-protein-coupled receptor pathways, are indicated and discussed further in the text. Question marks indicate that the role of these new players is not completely clear or that they have been reported to have an enhancing or reducing effect on the outcome measure depending on the publication. AKT, protein kinase B; cAMP, cyclic adenosine monophosphate; DENND1B, DENN domain-containing 1B; FIZZ1, found in inflammatory zone 1; GRB10, growth factor receptor bound protein 10; IL, interleukin; IL-13Rα1, interleukin-13 receptor α1; ILC2, type-2 innate lymphoid cells; IRS-2, insulin receptor substrate 2; mTORC, mammalian target of rapamycin complex; PI3-K, phosphoinositide 3-kinase; SNP, single nucleotide polymorphism; SOCS, suppressor of cytokine signaling; STAT6, signal transducer and activator of transcription 6; STUB1, STIPI homology and U-box-containing protein 1; TAS2R, taste 2 receptor; TGF-β, transforming growth factor-β; TH2, T helper type-2; TSLP, thymic stromal lymphopoietin; YKL-40, chitinase-3-like protein 1.

A better understanding of both canonical and newly appreciated pathways of regulation of allergic inflammation will inform the development of strategies to better control asthma and its consequences in Th2-high asthmatics. The new players that we have discussed in this review, such as the role of epithelial-derived cytokines in type-2 cytokine production (TSLP, IL-25, and IL-33), the identification of novel ligands for the IL-13 receptor IL-13Rα2, the role of E3 ubiquitin ligases in T cell differentiation, and the role of cellular metabolism in type-2 immune responses, highlight the tremendous complexity of the pathways and feedback loops that regulate IL-4/IL-13 production, their receptors, their signaling pathways, and their downstream consequences (summarized in Figure 1). Some of these effects are uncovered only in the setting of specific polymorphisms or the presence or absence of other host factors (age as well as gut and lung microbiome). An appreciation of this complexity reinforces the importance of careful phenotyping of asthma endotypes in each patient to direct the appropriate intervention. Our emerging understanding of the intricate web of factors that modify Th2 responses will provide new therapeutic avenues that could lead to the development of better, more effective strategies to treat and/or prevent this increasingly common disease.
